# Spectrophotometric and Smartphone-Assisted Determination of Phenolic Compounds Using Crude Eggplant Extract

**DOI:** 10.3390/molecules24234407

**Published:** 2019-12-02

**Authors:** M.A. Morosanova, A.S. Bashkatova, E.I. Morosanova

**Affiliations:** Analytical Chemistry Division, Chemistry Department, Lomonosov Moscow State University, 119234 Moscow, Russia

**Keywords:** crude eggplant extract, polyphenol oxidase, phenolic compounds, smartphone-assisted determination, total polyphenol content

## Abstract

In order to develop a simple, reliable and low cost enzymatic method for the determination of phenolic compounds we studied polyphenol oxidase activity of crude eggplant (*S. melongena*) extract using 13 phenolic compounds. Catechol, caffeic and chlorogenic acids, and l-DOPA have been rapidly oxidized with the formation of colored products. Monophenolic compounds have been oxidized at a much slower speed. Ferulic acid, quercetin, rutin, and dihydroquercetin have been found to inhibit polyphenol oxidase activity of crude eggplant extract. The influence of pH, temperature, crude eggplant extract amount, and 3-methyl-2-benzothiazolinone hydrazone (MBTH) concentration on the oxidation of catechol, caffeic acid, chlorogenic acid, and l-DOPA has been investigated spectrophotometrically. Michaelis constants values decrease by a factor of 2 to 3 in the presence of MBTH. Spectrophotometric (cuvette and microplate variants) and smartphone-assisted procedures for phenolic compounds determination have been proposed. Average saturation values (HSV color model) of the images of the microplate wells have been chosen as the analytical signal for smartphone-assisted procedure. LOD values for catechol, caffeic acid, chlorogenic acid, and l-DOPA equaled 5.1, 6.3, 5.8 and 30.0 µM (cuvette procedure), 12.2, 13.2, 13.2 and 80.4 µM (microplate procedure), and 23.5, 26.4, 20.8 and 120.6 µM (smartphone procedure). All the variants have been successfully applied for fast (4-5 min) and simple TPC determination in plant derived products and l-DOPA determination in model biological fluids. The values found with smartphone procedure are in good agreement with both spectrophotometric procedures values and reference values. Using crude eggplant extract- mediated reactions combined with smartphone camera detection has allowed creating low-cost, reliable and environmentally friendly analytical method for the determination of phenolic compounds.

## 1. Introduction

Phenolic compounds are a group of molecules that play an important role in human health due to their high biological activity. Some endogenous molecules, such as catecholamine neurotransmitters, and many compounds from consumed foods with antioxidant activity are phenolic compounds [1,2]. Monitoring catecholamines, such as l-dihydroxyphenylalanine (l-DOPA), in biological fluids and pharmaceuticals is a relevant task for treating neurodegenerative diseases [3]. Food antioxidant activity is linked to its total polyphenol content (TPC), which is often determined without analyzing individual antioxidant components. Different phenolic compounds can be chosen as a reference: caffeic [1,2], gallic [4,5,6,7], and chlorogenic acids [8], catechol [9], guaiacol [10], etc. Due to their role in human health the determination of phenolic antioxidants in plant-derived products and phenolic neurotransmitters in biological fluids requires simple, fast and cheap procedures, including screening tools.

Enzymatic systems, including biosensors, are an efficient analytical tool for phenolic compounds determination having such advantages as selectivity, sensitivity, reproducibility, high sample throughput, and simplicity in use. Polyphenol oxidase (PPO) demonstrates some advantages over other enzymes: the ability to catalyze oxidation reaction without any additional cofactors, oxidation of phenolic compounds in the presence of oxygen and good stability [11,12]. PPO is a bifunctional enzyme that catalyzes two different reactions: cresolase activity (the hydroxylation of monophenols to *o*-diphenols) and catecholase activity (the oxidation of *o*-diphenols to *o*-quinones). PPO is actively used for creating various biosensors for phenolic compounds determination, these biosensors show high selectivity, sensitivity, good stability and short reaction times [11].

The analytical use of crude plant extracts is a promising approach. Firstly, natural medium offers high stability of the enzymes and provides all the necessary cofactors. Secondly, the low cost of plant extracts compared to the purified enzymes provides more applications available. Also, the elimination of enzyme purification steps minimizes chemical reagents consumption and waste generation, subsequently achieving more environmentally friendly enzymatic analytical methods, i.e., “green analytical chemistry”. All these advantages translate into a significant interest of creating electrochemical and spectrophotometric biosensor systems based on crude plant extracts. Many works have been published describing the analytical use of such extracts with PPO activity: banana [4,9,13], sapota [3], jurubeba [14], sweet potato [15], cara [16].

Eggplant (*Solanum melongena*) is a very rich source of PPO [12,17,18]. Analytical application of eggplant PPO is only described for electrochemical determination. Crude eggplant extract (EE) is used for paracetamol determination [19] and whole eggplant pulp pieces are used for catechol determination [20]. Spectrophotometric analytical procedures using eggplant PPO are not described to date.

Another advantage of developing the spectrophotometric procedure is that color changes can be easily observed. Replacing a spectrophotometer by a smartphone camera can increase the simplicity and availability of the spectrophotometric procedure [21]. Various color models can be used to obtain a reliable analytical signal [22].

The aim of the present work was to study the oxidation of various phenolic compounds in presence of eggplant crude extract in order to determine possible analytes and develop simple, rapid and reliable procedures of their determination in various samples using spectrophotometer, microplate reader and smartphone camera for the detection of the analytical signal.

## 2. Results and Discussion

### 2.1. Eggplant Extract Polyphenol Oxidase Activity and Substrate Specificity

Previously, eggplant has been reported to have polyphenol oxidase activity [12,17,18]. Purified eggplant PPO was characterized by much lower cresolase activity than catecholase activity [12], in another study purified eggplant PPO lacked any cresolase activity [18]. The substrate specificity of purified eggplant PPO showed that it belonged to the catechol oxidase family (EC 1.10.3.1) as the maximum activity was observed with catechol or its derivative substrates [18].

Eggplant crude extract [17] and tissues [19,20] also demonstrate PPO activity. Catechol, 4-methylcatechol, caffeic, chlorogenic, 3,4-dihydroxyhydrocinnamic acids, and paracetamol were shown to be substrates of eggplant PPO. The eggplant tissue [19] showed activity towards monophenol substrate (paracetamol) on the contrary to purified eggplant PPO, which may be explained by the loss of some enzyme activity during purification procedure. Using a native enzyme preparation, such as crude extract, seems to be a perspective way to keep all the benefits of enzymatic analytical procedures and broaden the number of possible analytes. In the present work, we aimed to study EE activity towards broad spectrum of different poly- and monophenolic compounds oxidation to determine the possible analytes and propose analytical procedures. The following phenolic compounds were used: catechol, gallic, caffeic, ferulic, and chlorogenic acids, quercetin, rutin, dihydroquercetin, l-DOPA, l-tyrosine, paracetamol, phenol and *p*-nitrophenol.

The polyphenol oxidase activity of crude eggplant extract (EE), prepared in this study, was determined by comparing the reaction speed of catechol oxidation in the presence of EE and purified mushroom tyrosinase. EE was characterized by 160 ± 6 U/mL (n = 3, *P* = 0.95) polyphenol oxidase activity which is 2.5 times higher than for banana extract that we studied before [4]. Total protein content of EE was also determined (1.1 ± 0.3 mg/mL, *n* = 3, *P* = 0.95). When stored at −20 °C EE retained 75% of activity after 6 months, when stored at +4 °C EE retained 75% of activity after 3 days and 40% of activity after 2 months.

We investigated EE activity towards various phenolic compounds in order to develop analytical procedures for their fast determination. Catechol, caffeic and chlorogenic acids, and l-DOPA were rapidly oxidized in presence of EE with the formation of colored products. Their oxidation products had the following absorbance maxima: 410 nm for catechol, 420 nm for caffeic and chlorogenic acids, 480 nm for l-DOPA. Similarly to [18] in our work EE activity towards l-DOPA oxidation was lower than for other catechol derivatives. For other studied phenolic compounds (gallic acid, ferulic acid, quercetin, rutin, dihydroquercetin, l-tyrosine, paracetamol, phenol, *p*-nitrophenol) no colored oxidation products were observed in the presence of EE during the observation time (4 min).

Monophenolic compounds, such as paracetamol, were previously shown to interact with eggplant PPO [19]. We suggested that the oxidation reaction was much slower for monophenolic compounds because of cresolase reaction step, and indeed, some colored products were observed for paracetamol and l-tyrosine if the reaction mixture was left overnight. Such slow reaction speed was not acceptable for our analytical application, so we did not study monophenol substrates further.

Gallic acid was not oxidized in the presence of EE similarly to purified eggplant PPO [18]. Ferulic acid, quercetin, rutin, and dihydroquercetin interaction with eggplant PPO was studied for the first time. These compounds did not serve as substrates for EE and furthermore, these compounds were found to inhibit catechol oxidation by EE. Ferulic acid is a known PPO inhibitor [23] and quercetin is known to inhibit tyrosinase [24].

The substrate specificity study allowed us to select four phenolic compounds (catechol, caffeic acid, chlorogenic acid, and l-DOPA) in order to develop fast analytical procedures for their determination.

### 2.2. Eggplant Extract Polyphenol Oxidase Interaction with Catechol, Caffeic Acid, Chlorogenic Acid, and l-DOPA

In order to choose the reaction conditions we investigated the influence of pH and temperature on oxidation of catechol, caffeic acid, chlorogenic acid, and l-DOPA. The reaction speed was calculated as a difference between absorbance at 30 s and at 150 s after the reaction start (ΔA).

**Figure 1:**
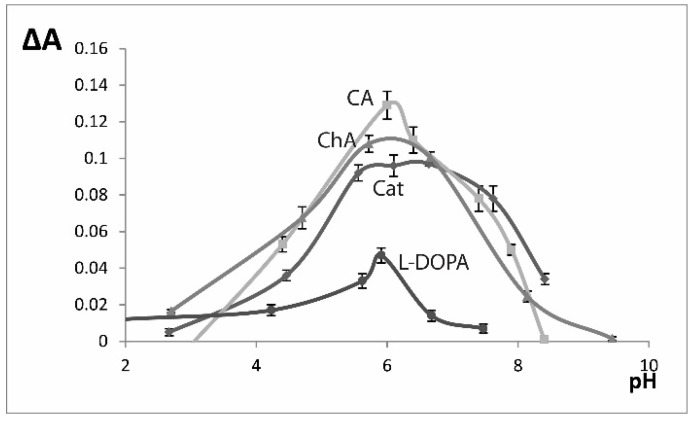
Influence of pH on the oxidation reaction speed (ΔA = A_150 s_ − A_30 s_). C(substrate) = 2.5 × 10^−4^ M. Cat - catechol (λ 410 nm), CA - caffeic acid (λ 420 nm), ChA - chlorogenic acid (λ 420 nm), l-DOPA (λ 480 nm). n = 3.

**Figure 2:**
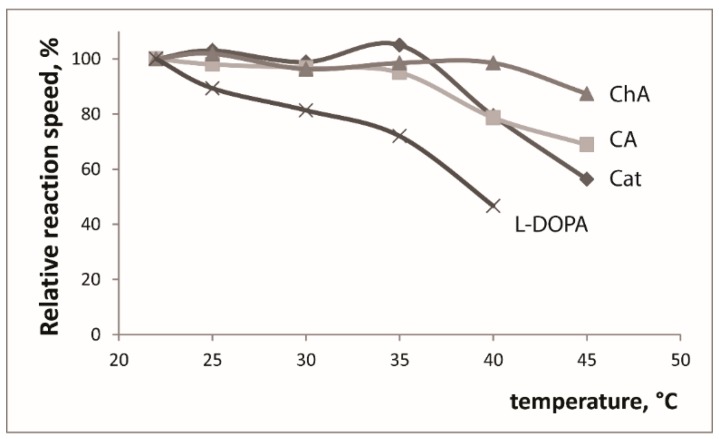
Influence of temperature on the relative oxidation reaction speed (ΔA = A_150 s_-A_30 s_), the reaction speed at room temperature is defined as 100%. C(substrate) = 2.5 × 10^−4^ M, pH 6. Cat - catechol (λ 410 nm), CA - caffeic acid (λ 420 nm), ChA - chlorogenic acid (λ 420 nm), l-DOPA (λ 480 nm).

**Figure 3:**
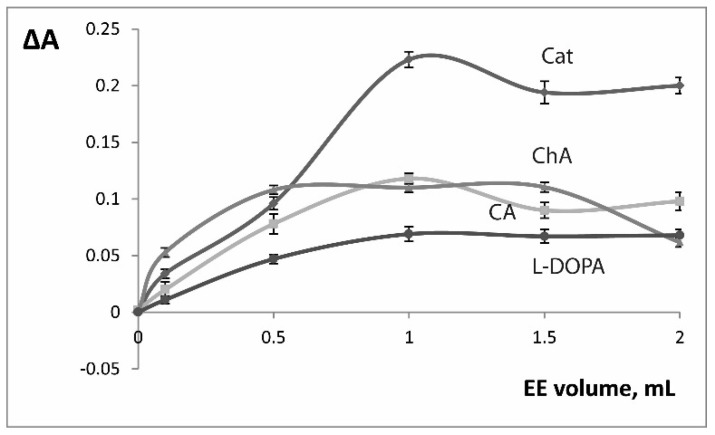
Influence of EE volume on the oxidation reaction rate (ΔA = A_150 s_ − A_30 s_). C(substrate) = 2.5 × 10^−4^ M, pH 6. Cat - catechol (λ 410 nm), CA - caffeic acid (λ 420 nm), ChA - chlorogenic acid (λ 420 nm), l-DOPA (λ 480 nm). n = 3.

The influence of pH on the reaction speed was studied in the range 2.0–9.0 (Figure 1), for all studied substrates the reaction speed reached maximal values in the 5.0–7.0 pH range. All the further activity measurements were conducted at pH 6.0. We studied the influence of temperature (22–45 °C): no increase of the reaction speed was detected and at temperatures higher than 35 °C reaction became significantly slower (Figure 2). We conducted all the further experiments at room temperature (22 °C). The influence of enzyme amount (EE volume) was also investigated and the 1.0 mL volume was chosen for further experiments (Figure 3).

3-Methyl-2-benzothiazolinone hydrazone (MBTH) is known to couple with the product of phenolic compounds oxidation (both enzymatic and non-enzymatic) and accelerate the reaction speed [4,25,26,27]. MBTH is widely used in optical biosensors based on laccase and tyrosinase enzymes [28,29,30,31,32]. The interaction of MBTH with the enzymatically produced quinones leads to optical signal amplification because the reaction equilibrium is shifted towards the product and the azo dye product is intensely colored.

**Figure 4:**
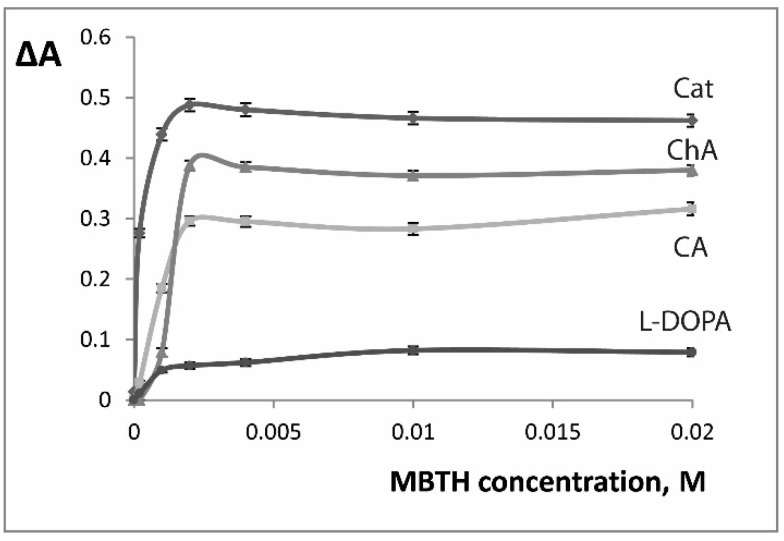
Influence of MBTH concentration on the oxidation reaction speed (ΔA = A_150 s_ − A_30 s_). C(substrate) = 2.5 × 10^−4^ M, pH 6. Cat - catechol (λ 460 nm), CA - caffeic acid (λ 520 nm), ChA - chlorogenic acid (λ 520 nm), l-DOPA (λ 520 nm). n = 3.

The influence of MBTH on eggplant PPO catalyzed oxidation was studied for the first time. In presence of MBTH the spectra of the products of enzymatic oxidation by EE demonstrated the following absorbance maxima: 460 nm for catechol, 520 nm for l-DOPA and caffeic and chlorogenic acids. The reaction speed increased with the increase of MBTH concentration (Figure 4) and 2.0 × 10^−3^ M concentration of MBTH was chosen for further experiments.

The enzymatic activity of EE towards catechol, caffeic acid, chlorogenic acid, and l-DOPA was studied in the broad range of substrate concentrations. The enzymatic oxidation reaction Michaelis constants (K_m_) and maximum rates (V_max_) were calculated using Lineweaver–Burk plots (Table 1). For catechol, caffeic and chlorogenic acids Michaelis constants had similar values, because the studied substrates have similar structure. The maximum value of V_max_ was observed for chlorogenic acid.

Kinetic parameters of all the studied phenolic compounds oxidation in presence of MBTH were determined using Lineweaver–Burk plots (Table 1). K_m_ values decreased by a factor of 2 to 3 for the studied substrates. V_max_ increased significantly only for catechol, which can be explained by its simple structure: V_max_ increases because of the azo dye formation which increases absorbance of the mixture, and the simpler is the structure of the quinone the easier it is to form the azo dye.

The effect of MBTH on the EE enzymatic oxidation (decrease of K_m_ values and increase of V_max_ values) is similar to that observed for other crude plant extracts [4]. K_m_ values decreased by a factor of 2 to 3 on the average for EE, which is a less pronounced effect than for banana extract (a factor of 12 on the average). However, this decrease was similar for all the studied substrates for both PPO extracts (eggplant and banana) compared to an uneven effect for peroxidase containing green bean extract (from 2 to 20 times K_m_ values decrease) [4]. This characteristic of PPO extracts makes them preferable for total phenol content determination procedures.

The proposed system (EE+MBTH) was used for the development of spectrophotometric procedures of phenolic compounds determination.

### 2.3. Analytical Application

Chosen reaction conditions were used to develop analytical procedures for phenolic compounds determination in three variants: cuvette, microplate and smartphone-assisted procedures. The initial reaction rate was estimated as the difference between the absorbance values at 270 s and 30 s and was used as analytical signal.

#### 2.3.1. Cuvette and Microplate Procedures with Spectrophotometer

The analytical ranges, limit of detection (LOD) values, and limit of quantitation (LOQ) values for EE and EE + MBTH cuvette procedures are given in Table 2. For all the studied compounds LOD values decreased approximately by one order of magnitude in the presence of MBTH. LOD values were similar for all the studied compounds which can be useful for TPC determination (Table 2).

**Figure 5:**
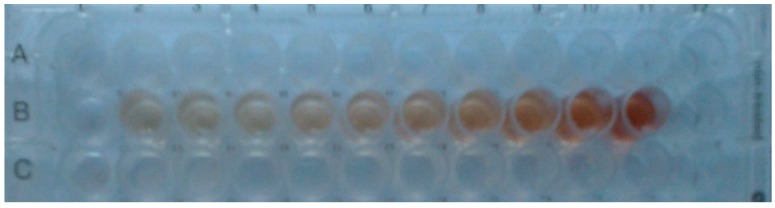
Microplate assay for phenolic compounds determination: left to right 0, 0, 0, 1.6·10^−5^ M, 3.1·10^−5^ M, 6.3·10^−5^ M, 1.3·10^−4^ M, 2.5·10^−4^ M, 5.0·10^−4^ M, 1.0·10^−3^ M catechol.

The EE + MBTH system was chosen for microplate procedure development. Microplate assays allow fast and simultaneous determination of many samples. Both reagents amount and time needed for the analysis is reduced, which makes microplate assays a very useful tool for applied analytical procedures. To create the microplate assay we adjusted the earlier developed procedure so that the total volume of the reaction mixture was 200 µL while the proportionality was preserved. Calibration curves were constructed for catechol, caffeic acid, chlorogenic acid, and l-DOPA (Figure 5 shows the calibration for catechol). The analytical ranges, limit of detection (LOD) values, and limit of quantitation (LOQ) values are given in Table 2.

One of the applications of TPC determination is food and drinks analysis. Ascorbic, citric, and tartaric acids, sulfite, sucrose and ethanol are often present in food and drinks as natural ingredients or conservatives.

**Figure 6:**
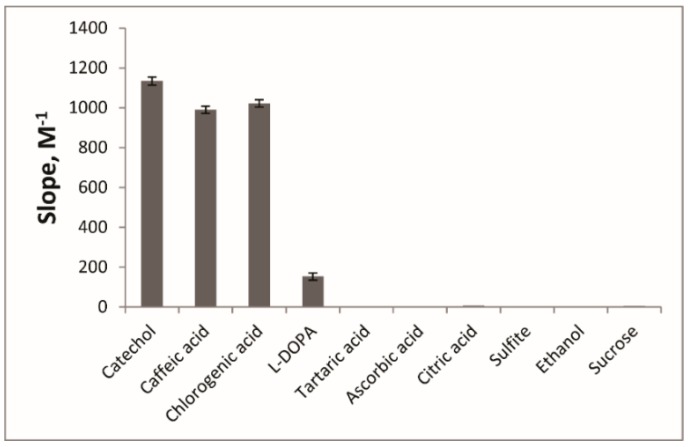
Calibration curve slopes for analytes and interfering compounds in the EE + MBTH system (cuvette procedure). pH 6, λ 520 nm.

We used cuvette procedure to investigate the influence of these potentially interfering substances on the TPC determination in plant derived products. The slope values for analytes and interfering compounds are given in the Figure 6.

Catechol, caffeic acid and chlorogenic acid have very similar slope values which is important for TPC determination. l-DOPA calibration curve was characterized by lesser slope value as it was observed for purified eggplant PPO earlier [18].

The interfering substances did not produce any analytical signal, so the next step was to study their influence on the determination of analytes. Ascorbic, citric and tartaric acids and sulfite ions are well-known PPO inhibitors [4]. Ethanol was used for eggplant fruits preservation [33], so it can also be considered as potent PPO inhibitor. The interfering substances thresholds were determined as a concentration that gives 10% error in the determination of 1.0·10^−3^ M catechol using EE+MBTH procedure (Table 3). Citric acid and sulphite thresholds are similar to those observed for previously studied extracts [4], and the threshold for ascorbic acid is 10 times higher. This is an important achievement as ascorbic acid is an ubiquitous substance in plant derived food products.

Both cuvette and microplate analytical procedures were used for TPC determination in tea and green coffee extract tablets (Table 4). The results of the determination were compared with an independent method and good agreement was observed.

Both cuvette and microplate analytical procedures were used for l-DOPA determination in spiked samples of synthetic serum and urine (Table 4). The results of recovery test show that the components of synthetic serum and urine did not interfere l-DOPA determination (recoveries were in the 96–108% range).

The analytical ranges of the developed procedures allowed TPC determination in beverages and pharmaceuticals and l-DOPA determination in biological fluids. For cuvette procedure LOD values were as low as (0.5–1.1)·10^−5^ M. They are similar to our previous results [4] and comparable with other spectroscopy based biosensors (Table 5) which allows reliable determination of catechol, caffeic acid, chlorogenic acid, and l-DOPA in a broad range of samples. The sensitivity of the determination is a little decreased when using microplate assay procedure. On the other hand, the microplate assay procedure was characterized by 20 times less required reagents volume and much shorter time of analysis (up to 90 times faster). Both developed procedures can serve as a tool for fast and simple TPC and l-DOPA determination.

#### 2.3.2. Smartphone-Assisted Microplate Procedure

The widespread availability of smartphone and rapidly growing communications infrastructure in the world represents a great opportunity for reading and sharing diagnostic or analytical data [34]. Microplate assays are easily combined with smartphones [35] and this allows simultaneous calibration and analyte determination like in standard microplate-based ELISA. The image shown in Figure 5 was taken with a smartphone camera and demonstrates a color change visible even to a naked eye. We used free GIMP software to process such photos and develop the analytical procedure for phenolic compounds determination.

Different approaches to replace a spectrophotometer by digital camera-like devices (digital cameras, smartphone cameras, office scanners) are being widely used due to the increasing availability of such devices. Measuring the light after it passes through a colored probe can be done using an additional light source (a lamp, light-emitting display, smartphone flash light) or ambient light reflection from a colored or white surface [21]. Various color models (RGB, HSV/HSL, CIELab) values or their derivatives can be used as an analytical signal [22]. Although the most popular model remains RGB, the HSV model has been shown to be a more relevant representation of colors wavelength [36]. The H values (hue) of HSV model have been used as an analytical signal when the quantification of the analyte was based on the change in the color [37]. The S values (saturation) of HSV model have been shown to have the strong correlation with the absorbance values in the case where the base color remains the same and its intensity varies [35].

The images of the microplates with calibration curves for catechol, caffeic acid, chlorogenic acid, and l-DOPA were taken with a smartphone camera after their absorbance was measured for 4 min in the spectrophotometer. We measured the average S values for each well and calculated Pearson correlation coefficients between S values and absorbance values. The coefficients equaled 0.9987, 0.9884, 0.9784, and 0.9800 for catechol, caffeic acid, chlorogenic acid, and l-DOPA calibration curves, respectively. Such a strong correlation indicates that S values can be used as an analytical signal for the developed microplate procedure. The analytical ranges, limit of detection (LOD) values, and limit of quantitation (LOQ) values were calculated using S values (Table 2) and almost no loss of sensitivity was observed comparing to the microplate procedure. The smartphone procedure was also applied to the real objects analysis (Table 4) and it was characterized by 9–18% RSD values and 92–120% recoveries. The results present a very simple, low cost and reliable procedure for the determination of phenolic compounds in various complex matrices.

The real sample analysis results obtained by the application of cuvette, microplate and smartphone-assisted procedures were compared to each other and to reference values using Tukey’s test. Green tea sample, green coffee extract tablets sample, synthetic serum sample 2, and synthetic urine sample 1 (Table 4) were used in this comparison. No significant difference was found between the reference values and values found by cuvette, microplate and smartphone-assisted procedures for any of the samples (*p* > 0.1, Tables S1–S4). The important result of this Tukey’s test application is that the values found with smartphone-assisted procedure are in good agreement with both spectrophotometric procedures values and with reference values, which means that the smartphone-assisted procedure provides reliable results.

## 3. Materials and Methods

### 3.1. Reagents and equipment

The following reagents were purchased from Acros Organics (Morris Plains, NJ, USA): sodium carbonate, copper(II) sulfate, tartaric acid, sodium hydroxide, citric acid, ascorbic acid, sodium sulfite, catechol, gallic, caffeic, chlorogenic, and ferulic acids, quercetin, rutin, dihydroquercetin, l-dihydroxyphenylalanine, l-tyrosine, paracetamol, phenol, *p*-nitrophenol and 3-methyl-2-benzothiazolinone hydrazone (MBTH). All the reagents were of analytical grade. Stock solutions of phenolic compounds (1.0·× 10^−2^ M) were prepared with doubly distilled water. Only freshly prepared solutions of phenolic compounds were used. 3-methyl-2-benzothiazolinone hydrazone stock solution (1.0 ×·10^−3^ M) was prepared with doubly distilled water and stored at +4 °C.

Phosphate buffer solutions, oxalate buffer solution, phthalate buffer solution, and borate buffer solution were used. pH of the reaction mixtures was measured with HI83303 photometer/pH-meter and HI11310 pH electrode (Hanna Instruments, Woonsocket, RI, USA).

Mushroom tyrosinase from *Aspergillus niger* (CAS 9002-10-2, 3900 U/mg) was purchased from Sigma (St. Louis, MO, USA). Bovine serum albumin (BSA) was purchased from PanEco (Moscow, Russia.

Spectra of colored products of enzymatic oxidation of phenolic compounds were recorded with SPECTROstar Nano spectrophotometer (BMG Labtech, Ortenberg, Germany) which is compatible with both cuvettes and microplates and has a built-in temperature incubator. Spectra were analyzed with MARS 3.20 software (BMG Labtech, Ortenberg, Germany) and statistical analysis was carried out using MS Excel 2010 (Microsoft, Redmond, WA, USA).

For the smartphone-assisted determination the images of the microplates were taken with an Alcatel POP 2 5042D smartphone and analyzed with GIMP 2.8.4 software (Free Software Foundation, Cambridge, MA, USA).

### 3.2. Crude Eggplant Extract Preparation

In the present work the procedure for plant extract preparation was adapted from [4]: eggplant extract was prepared by stirring homogenized eggplant pulp (100.0 g, purchased from local markets) in 0.07 M phosphate buffer (pH 7, 200.0 mL) at 0 °C for 30 min, then filtering twice through paper filter. One mL aliquots of extract were stored at −20 °C until use.

The activity of the crude extract used in this work has been determined by comparing the reaction rate of catechol oxidation in the presence of the crude extract and mushroom tyrosinase as in [4]. We conducted all the experiments at room temperature (22 °C). The activity of the extracts remains unchanged after at least 6 months of storage at −20 °C.

Total protein measurements were performed by the Biuret method as in [4]. The Biuret reagent was prepared by dissolution of copper(II) sulfate, sodium potassium tartrate, and sodium hydroxide in distilled water. Bovine serum albumin stock solution (2.5 mg/mL) was prepared with doubly distilled water. Calibration curve was prepared by mixing 2.0 mL of BSA solution in the 0.1–2.5 mg/mL concentration range with 1.0 mL of Biuret reagent; blank was prepared by mixing 2.0 mL of doubly distilled water with 1.0 mL of Biuret reagent. The absorbance was measured at 540 nm. The total protein content in plant crude extracts was determined in triplicate.

### 3.3. General Procedure for Crude Plant Extract and Phenolic Compounds Interaction Study

The following phenolic compounds were studied: catechol, gallic, caffeic, ferulic, and chlorogenic acids, quercetin, rutin, dihydroquercetin, l-DOPA, l-tyrosine, paracetamol, phenol, *p*-nitrophenol. Two mL of phenolic compound solution (1.0·10^−6^–1.0·10^−2^ M) was mixed with 0.5 mL of buffer solution (pH 7), 0.5 mL of crude eggplant extract, and the spectra of the colored reaction products were recorded for 4 min. To study the possible inhibitory action of the studied phenolic compounds 2.0 mL of 5.0·× 10^−3^ M catechol solution was added.

For catechol, caffeic acid, chlorogenic acid, and l-DOPA the influence of the reaction conditions were studied: 2.0 mL of phenolic compound solution (1.0·× 10^−6^–1.0·× 10^−2^ M) was mixed with 0.5 mL of buffer solution (pH 2–8), 0.1–1.5 mL of crude eggplant extract and the spectra of the colored reaction products were recorded for 4 min. Total volume of the probe remained 4.0 mL. To study the effect of MBTH on the enzymatic oxidation of 0.5 mL of MBTH (2.0 ×·10^−4^–2.0 ×·10^−2^ M) was added to the reaction mixture prior to the crude eggplant extract addition. Michaelis constants (K_m_) and maximum rates (V_max_) for catechol, caffeic acid, chlorogenic acid, and l-DOPA were calculated using Lineweaver–Burk plots.

### 3.4. Samples Preparation

1.0 g of tea sample was boiled in 100.0 mL of distilled water. After cooling the samples were filtered. Filtered tea extracts samples were diluted to the mark of 100.0 mL with distilled water. Green coffee extract tablets were ground and 50 mg of the powder was added to 100 mL of distilled water. After mixing for 30 min, the solution was filtered and diluted to the mark of 100.0 mL. Synthetic serum was prepared as in [38]; synthetic urine was prepared as in [39].

### 3.5. Procedures of Phenolic Compounds Determination

For the cuvette procedure 2.0 mL of sample was mixed with 0.5 mL of phosphate buffer (pH 6), 0.5 mL of MBTH (2.0 ×·10^−3^ M). Then 1.0 mL of crude eggplant extract was added and the absorbance was measured at 30 s and 270 s at 520 nm. The difference of the absorbance values was used as analytical signal. TPC in tea samples was determined in triplicate using a calibration curve of caffeic acid in the 1.9·× 10^−5^ to 5.0·× 10^−4^ M range. TPC in tablets samples was determined in triplicate using a calibration curve of chlorogenic acid in the 1.4·× 10^−5^ to 5.0·× 10^−4^ M range. l-DOPA concentration in synthetic biological fluids was determined in triplicate using a calibration curve of l-DOPA in the 3.3·× 10^−5^ to 1.3·× 10^−3^ M range.

For the microplate procedure 96-well microplates wells were filled with 50 µL 1.0·× 10^−3^ M MBTH buffered solution (phosphate buffer, pH 6), then 100 µL of either sample or standard solution was added. After adding 50 µL of crude eggplant extract to each well the plate was introduced into SPECTROstar Nano spectrophotometer and shaken for 10 s to start the reaction. The absorbance at 520 nm was measured for 4 min, the well with 200 µL of distilled water was used as blank.

The images of microplates against a white sheet of paper at approximately 5 min after reaction start were taken with an Android smartphone camera (5 MP resolution). The resulting JPG images were analyzed with free GIMP 2.8.4 software to get the average color saturation (S, %) for each well using the HSV color model and the Color Picker tool with the radius adjusted to the well size on the image.

Folin-Ciocalteau assay for TPC determination was conducted similarly to [4]. The TPC in samples was determined using a standard curve of caffeic acid ranging from 2.7 ×·10^−5^ to 2.0·× 10^−4^ M. TPC was expressed as caffeic acid equivalents (CAE) in mg/g for tea.

The limit of detection (LOD) was calculated as 3·standard deviation of the blank absorbance (n = 3) divided by the slope value. The limit of quantitation (LOQ) was calculated as 3·LOD.

## 4. Conclusions

In the present work, we investigated the polyphenol oxidase activity of eggplant crude extract (EE) towards 13 phenolic compounds: catechol, gallic, caffeic, ferulic, and chlorogenic acids, quercetin, rutin, dihydroquercetin, l-DOPA, l-tyrosine, paracetamol, phenol, *p*-nitrophenol. Catechol, caffeic acid, chlorogenic acid, and l-DOPA have been rapidly oxidized in presence of EE with the formation of colored products. Monophenolic compounds have been oxidized at a much slower rate. Ferulic acid, quercetin, rutin, and dihydroquercetin have been found to inhibit catechol oxidation in the presence of EE.

The effect of 3-methyl-2-benzothiazolinone hydrazone (MBTH) on catechol, caffeic acid, chlorogenic acid, and l-DOPA oxidation has been studied: MBTH increased substrates affinity and reaction speed, allowing more sensitive determination. Spectrophotometric procedures using EE and MBTH have been proposed for the determination of total polyphenol content in caffeic acid equivalents, or chlorogenic acid equivalents in plant derived products, and for the determination of l-DOPA in biological fluids. The proposed procedures are characterized by higher interference thresholds comparing to previously studied crude plant extracts [4]. Detection limits are in the (0.5–3.0)·10^−5^ M range.

A microplate assay variant has been developed for the EE + MBTH procedure using 96-well microplates. This assay needs only 100 µL of sample and allows high-throughput screening up to 30 samples in 4 min. The sensitivity of the determination is slightly decreased compared to cuvette procedure, because of lesser optical length. The detection limits increased by the factor of approximately 2.5 for all the studied analytes. The developed assay can serve as a valuable screening tool for the applications like TPC determination in food and pharmaceuticals.

The combination of the microplate assay with smartphone-assisted detection has been proposed for the development of even simpler and cheaper procedure. The images of microplates have been analyzed with free GIMP software and average saturation values have been chosen as an analytical signal. The resulting analytical procedures have been characterized by (2.1–12.1)·10^−5^ M LOD values and have been applied to phenolic compounds determination in food, pharmaceuticals and biological fluids. The procedure using crude plant extract and smartphone camera detection has been proposed for the first time.

## Figures and Tables

**Table 1:** Kinetic parameters of enzymatic oxidation of catechol, caffeic acid, chlorogenic acid, and l-DOPA in the presence of eggplant extract in the absence of MBTH and in the presence of 2.0 × 10^−3^ M MBTH.

Substrate	Eggplant Extract		Eggplant Extract + MBTH	
	V_max_, min^−1^	K_m_, M	V_max_, min^−1^	K_m_, M
Catechol	0.167	1.3∙10^−3^	0.561	3.9∙10^−4^
Caffeic acid	0.315	1.6∙10^−3^	0.317	4.8∙10^−4^
Chlorogenic acid	0.496	1.4∙10^−3^	0.577	6.4∙10^−4^
l-DOPA	0.115	8.6·10^−4^	0.131	5.1∙10^−4^

**Table 2:** Analytical parameters of the cuvette and microplate procedures using eggplant crude extract in absence of MBTH and in presence of 1.0 × 10^−3^ M MBTH (n = 3 for LOD and LOQ).

Substrate	Spectrophotometric Determination									Smartphone Determination		
	Eggplant Extract (Cuvette)			Eggplant Extract + MBTH (Cuvette)			Eggplant Extract + MBTH (Microplate)			Eggplant Extract + MBTH (Microplate)		
	Analytical Range, M∙10^5^	LOD, M∙10^5^	LOQ, M∙10^5^	Analytical Range, M∙10^5^	LOD, M∙10^5^	LOQ, M∙10^5^	Analytical Range, M∙10^5^	LOD, M∙10^5^	LOQ, M∙10^5^	Analytical Range, M∙10^5^	LOD, M∙10^5^	LOQ, M∙10^5^
Catechol	14.3–125.0	4.8	14.3	1.5–125.0	0.5	1.5	3.6–50.0	1.2	3.6	7.2–50.0	2.4	7.2
Caffeic acid	12.0–100.0	4.0	12.0	1.9–50.0	0.6	1.9	3.9–50.0	1.3	3.9	7.8–50.0	2.6	7.8
Chlorogenic acid	9.5–50.0	3.2	9.5	1.4–50.0	0.5	1.4	3.9–25.0	1.3	3.9	6.3–25.0	2.1	6.3
l-DOPA	22.8–125.0	7.6	22.8	9.0–125.0	3.0	9.0	24.0–100.0	8.0	24.0	36.3–100.0	12.1	36.3

**Table 3:** Interference thresholds for the determination of 1.0 ×·10^−3^ M catechol (cuvette procedure).

Interfering Substance	Threshold (Concentration Causing 10% Error)
Ascorbic acid	2.0·10^−4^ M
Citric acid	1.8·10^−3^ M
Tartaric acid	1.8·10^−3^ M
Sulfite	1.9·10^−4^ M
Ethanol	7.0 %vol.

**Table 4:** Application of cuvette, microplate and smartphone procedures for the analysis of various samples (n = 3, *P* = 0.95).

Analyte	Sample	Found With Cuvette Procedure (RSD, %)	Found With Microplate Procedure (RSD, %)	Found With Smartphone Procedure (RSD, %)	Reference Value (RSD, %)
TPC in CA equivalents	Green tea	31.0 ± 5.3 mg/g (10.1)	30.8 ± 7.3 mg/g (14.0)	38.3 ± 11.3 mg/g (17.6)	31.9 ± 1.3 mg/g (2.4)^a^
	Black tea	25.5 ± 2.7 mg/g (6.2)	-	-	26.3 ± 1.8 mg/g (4.0)^a^
TPC in ChA equivalents	Green coffee extract tablets	136 ± 10 mg/tablet (5.4)	140 ± 19 mg/tablet (9.9)	120 ± 20 mg/tablet (11.8)	130 mg/tablet^b^
Caffeic acid	Synthetic urine sample	-	(1.1 ± 0.1)·10^−4^ M (5.4)	(1.2 ± 0.2)·10^−4^ M (8.7)	1.0·10^−4^ M^c^
l-DOPA	Synthetic serum sample 1	(2.4 ± 0.5)·10^−4^ M (12.9)	-	-	2.5·10^−4^ M^c^
	Synthetic serum sample 2	(5.2 ± 0.3)·10^−4^ M (2.9)	(5.0 ± 0.7)·10^−4^ M (8.6)	(4.9 ± 1.1)·10^−4^ M (12.7)	5.0·10^−4^ M^c^
	Synthetic urine sample 1	(5.4 ± 0.4)·10^−4^ M (4.7)	(4.9 ± 0.8)·10^−4^ M (10.5)	(5.4 ± 1.2)·10^−4^ M (13.3)	5.0·10^−4^ M^c^
	Synthetic urine sample 2	(9.7 ± 0.8)·10^−4^ M (5.0)	-	-	1.0·10^−3^ M^c^

^a^ FC method, ^b^ data given by the producer, ^c^ added amount.

**Table 5:** Comparison of spectrophotometric enzymatic procedures of phenolic compounds determination.

Enzyme	Time of Analysis, min	Analyte	LOD, µM	Reference
Laccase	10	Catechol	330	[29]
Tyrosinase	15	4-Chlorophenol	0.9	[31]
		Phenol	1.0	
		*m*-Cresol	1.0	
		*p*-Cresol	3.0	
Tyrosinase	2	l-DOPA	23	[32]
Horseradish peroxidase	5	Guaiacol	10	[28]
		Resorcinol	5	
		*o*-Cresol	12	
Banana crude extract	1	Catechol	4.6	[4]
		Gallic acid	29	
Green bean crude extract	1	Caffeic acid	5.0	
		Ferulic acid	3.9	
		Quercetin	1.0	
Eggplant crude extract	4	Catechol	5.1	Present work(cuvette variant)
		Caffeic acid	5.3	
		Chlorogenic acid	4.6	
		l-DOPA	30.0	

## Supplementary Materials

The following are available online, Tables S1–S4: Tukey’s test results.

## Abbreviations

BSA	Bovine serum albumin
CA	Caffeic acid
Cat	Catechol
ChA	Chlorogenic acid
l-DOPA	l-Dihydroxyphenylalanine
EE	Crude eggplant extract
HSV	Hue saturation value
LOD	Limit of detection
LOQ	Limit of quantification
MBTH	3-methyl-2-benzothiazolinone hydrazone
PPO	Polyphenol oxidase
TPC	Total polyphenol content
