# Supplementary material for: Spectrophotometric and Smartphone-Assisted Determination of Phenolic Compounds Using Crude Eggplant Extract
Source: Molecules. 2019 Dec 2;24(23):4407. doi: 10.3390/molecules24234407 (PMC6930448; doi:10.3390/molecules24234407)
Supplement: Supplementary file 1 [file molecules-24-04407-s001.zip › supplementary files/abbreviations table.docx]

| BSA | Bovine serum albumin |
| --- | --- |
| CA | Caffeic acid |
| Cat | Catechol |
| ChA | Chlorogenic acid |
| L-DOPA | L-dihydroxyphenylalanine |
| EE | Crude eggplant extract |
| HSV | Hue saturation value |
| LOD | Limit of detection |
| LOQ | Limit of quantification |
| MBTH | 3-methyl-2-benzothiazolinone hydrazone |
| PPO | Polyphenol oxidase |
| TPC | Total polyphenol content |

Table of the abbreviations
