# Supplementary material for: Spectrophotometric and Smartphone-Assisted Determination of Phenolic Compounds Using Crude Eggplant Extract
Source: Molecules. 2019 Dec 2;24(23):4407. doi: 10.3390/molecules24234407 (PMC6930448; doi:10.3390/molecules24234407)
Supplement: Supplementary file 1 [file molecules-24-04407-s001.zip › supplementary files/supplementary tables.docx]

Table S1. Tukey’s test p-values for the comparison of TPC values found in green tea sample. A – cuvette procedure results, B – microplate procedure results, C – smartphone-assisted procedure results, D – reference values (n=3).

| Pair | Tukey’s test p-value |
| --- | --- |
| A vs B | 0.90 |
| A vs C | 0.28 |
| A vs D | 0.90 |
| B vs C | 0.26 |
| B vs D | 0.90 |
| C vs D | 0.38 |

Table S2. Tukey’s test p-values for the comparison of TPC values found in green coffee extract tablets sample. A – cuvette procedure results, B – microplate procedure results, C – smartphone-assisted procedure results, D – reference values (n=3).

| Pair | Tukey’s test p-value |
| --- | --- |
| A vs B | 0.90 |
| A vs C | 0.22 |
| A vs D | 0.85 |
| B vs C | 0.13 |
| B vs D | 0.65 |
| C vs D | 0.54 |

Table S3. Tukey’s test p-values for the comparison of L-DOPA concentration values found in spiked synthetic serum sample. A – cuvette procedure results, B – microplate procedure results, C – smartphone-assisted procedure results, D – reference values (n=3).

| Pair | Tukey’s test p-value |
| --- | --- |
| A vs B | 0.71 |
| A vs C | 0.60 |
| A vs D | 0.78 |
| B vs C | 0.90 |
| B vs D | 0.90 |
| C vs D | 0.90 |

Table S4. Tukey’s test p-values for the comparison of L-DOPA concentration values found in spiked synthetic serum sample. A – cuvette procedure results, B – microplate procedure results, C – smartphone-assisted procedure results, D – reference values (n=3).

| Pair | Tukey’s test p-value |
| --- | --- |
| A vs B | 0.56 |
| A vs C | 0.90 |
| A vs D | 0.69 |
| B vs C | 0.55 |
| B vs D | 0.90 |
| C vs D | 0.68 |
